# Mechanochemical Synthesis of MOF-303 and Its CO_2_ Adsorption at Ambient Conditions

**DOI:** 10.3390/molecules29112698

**Published:** 2024-06-06

**Authors:** Sylwia Głowniak, Barbara Szczęśniak, Jerzy Choma, Mietek Jaroniec

**Affiliations:** 1 Institute of Chemistry, Military University of Technology, 00-908 Warsaw, Poland; sylwia.glowniak@wat.edu.pl (S.G.); barbara.szczesniak@wat.edu.pl (B.S.); jerzy.choma@wat.edu.pl (J.C.); 2 Department of Chemistry and Biochemistry & Advanced Materials and Liquid Crystal Institute, Kent State University, Kent, OH 44242, USA

**Keywords:** metal–organic frameworks, mechanochemistry, CO_2_ adsorption

## Abstract

Metal–organic structures have great potential for practical applications in many areas. However, their widespread use is often hindered by time-consuming and expensive synthesis procedures that often involve hazardous solvents and, therefore, generate wastes that need to be remediated and/or recycled. The development of cleaner, safer, and more sustainable synthesis methods is extremely important and is needed in the context of green chemistry. In this work, a facile mechanochemical method involving water-assisted ball milling was used for the synthesis of MOF-303. The obtained MOF-303 exhibited a high specific surface area of 1180 m^2^/g and showed an excellent CO_2_ adsorption capacity of 9.5 mmol/g at 0 °C and under 1 bar.

## 1. Introduction

Global warming due to high anthropogenic CO_2_ emissions is acknowledged as a crucial global climate change issue. To tackle the problem, diverse CO_2_ capture technologies have been examined and introduced, including adsorption on porous materials, which became a favorable strategy. Metal–organic frameworks (MOFs) have been considered highly efficient porous adsorbents due to their outstanding properties, i.e., high porosity, flexibility, crystallinity, and good chemical and thermal stability [1,2]. MOFs are three-dimensional porous crystalline materials consisting of metal ions or clusters and organic linkers connected via coordination bonds. An extensive range of metal nodes and linkers creates almost unlimited opportunities for creating various porous metal–organic structures. Adsorption properties of MOFs depend on their porosity, characterized by the pore volume (V_t_), pore size distribution (PSD), specific surface area (SSA), and chemical composition (e.g., accessible metal sites and functionalized organic ligands). The scientific literature reports that MOFs show record amounts of adsorbed carbon dioxide. For instance, under high pressure of 50 bar, the CO_2_ adsorption capacity of MOF-210 was as high as 54.5 mmol/g [3], whereas, under atmospheric pressure, Cu- or Mg-containing MOFs (e.g., CuBTC and MOF-74(Mg)) are ones of the most efficient CO_2_ adsorbents [1,4].

**Figure 1:**
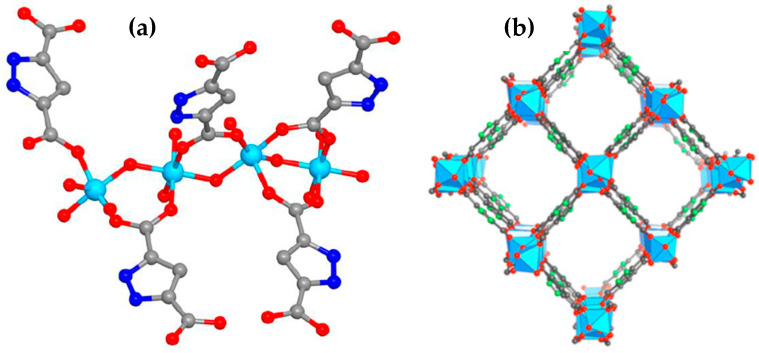
(**a**) Representation of the single-crystal structure of MOF-303. Hydrogen atoms have been excluded for clarity. Color scheme: C—gray, N—blue, O—red, Al—cyan. (**b**) Crystal structure of MOF-303 assembled from rod-like Al(OH)(COO)_2_ SBUs linked by HPDC linkers, forming an extended framework structure (xhh topology) with a 1D pore system. C—gray, N—green, O—red, Al—blue polyhedra. Adapted with permission from ref. [5]. Copyright © 2018 Creative Commons Attribution-NonCommercial License 4.0 (CC BY-NC).

Thousands of MOF structures have been successfully synthesized so far, including archetypal structures (e.g., ZIF-8 and CuBTC) as well as new, recently discovered MOFs. One of the relatively new MOFs is MOF-303, denoted as Al(OH)(HPDC), where HPDC stands for 1H-pyrazole-3,5-dicarboxylate. The crystal lattice of MOF-303 consists of rod-like Al(OH)(COO)_2_ secondary building units (SBUs) interconnected by HPDC linkers, forming an extended framework with a one-dimensional (1D) pore system with a pore size of 0.6 nm (Figure 1) [5].

MOF-303 was first synthesized in 2018 by the Yaghi group [5], and so far, there are not many papers devoted to this MOF. Moreover, studies on MOF-303 have been mainly dedicated to atmospheric water harvesting (AWH) [5,6,7,8,9,10,11,12,13,14,15,16,17,18,19,20], and this MOF has been identified as one of the most efficient mediums for this application [21]. It quickly turned out that it is also a very good adsorbent for CO_2_ [22], SO_2_ [23], NH_3_ [24], C_2_H_2_ [25], Xe [26], alcohols [27], particulate matters [28], water contaminants [29,30,31,32,33,34,35], separation [36,37,38,39,40,41,42,43,44], and other applications [45,46,47,48,49,50]. For example, MOF-303 has a high CO_2_ adsorption capacity of 5.1 mmol/g at 25 °C and under 1 bar, and what is equally important, the capacity was maintained after over 50 cycles of CO_2_ adsorption and desorption experiments [22]. Note that this adsorption capacity value exceeds the best values obtained for ultramicroporous carbons, which have been considered highly effective adsorbents for CO_2_ capture at ambient conditions [51]. As far as the authors know, the SSA of MOF-303 ranges from about 600 m^2^/g [52] to even 1529 m^2^/g [22], depending on the synthesis method, including the purification step [12,52].

Numerous efforts have been made to make the synthesis of porous materials (including CO_2_ adsorbents) as environmentally friendly as possible. Despite various synthetic procedures reported so far, most of them require hazardous organic solvents and generate large amounts of waste by-products that need to be remediated and/or recycled. Considering the mass production of MOFs, ecological and economic aspects are of high importance. Therefore, the development of alternative methods for the efficient and environmentally friendly synthesis of MOFs is required. Mechanochemical synthesis is established as a green process due to the significantly reduced consumption of solvents and liquid waste generation, as well as energy- and cost-effectiveness, simplicity, and timesaving. The mechanochemical synthesis relies on grinding/milling the mixture of solid precursors in a ball miller under solvent-free conditions (neat grinding, NG) or with a small amount of added liquids (liquid-assisted grinding, LAG) or with the addition of both salts and liquid (ion- and liquid-assisted grinding, ILAG). Despite a long history of mechanochemistry, the mechanochemical synthesis of MOFs started only in 2006 [53,54]. In recent years, publications have emerged on the preparation of various MOFs via water-assisted ball milling strategies [55,56,57,58,59,60]. Among an enormous number of known MOFs, as reported in [22], only about 25 of them show a CO_2_ adsorption capacity exceeding 5 mmol/g at 25 °C and under 1 bar. Thus, further studies on the mechanochemical synthesis of such MOFs, which are greener and feasible, are highly desirable.

Herein, we compared the physicochemical properties of MOF-303, ZIF-8, and CuBTC prepared via solvent-based and mechanochemical methods. Among these three MOFs, two of them, MOF-303 and CuBTC, show a very high CO_2_ uptake at 25 °C and under 1 bar, and MOF-303, to the best of our knowledge, was not synthesized yet via the mechanochemical method. Another popular MOF, ZIF-8, was also prepared using these two synthesis methods and characterized for the purpose of comparison. The results show that the morphology, crystallinity, porous structure, and adsorption properties of MOF strongly depend on its synthesis conditions.

## 2. Results and Discussion

### 2.1. Morphological and Structural Characterization

**Figure 2:**
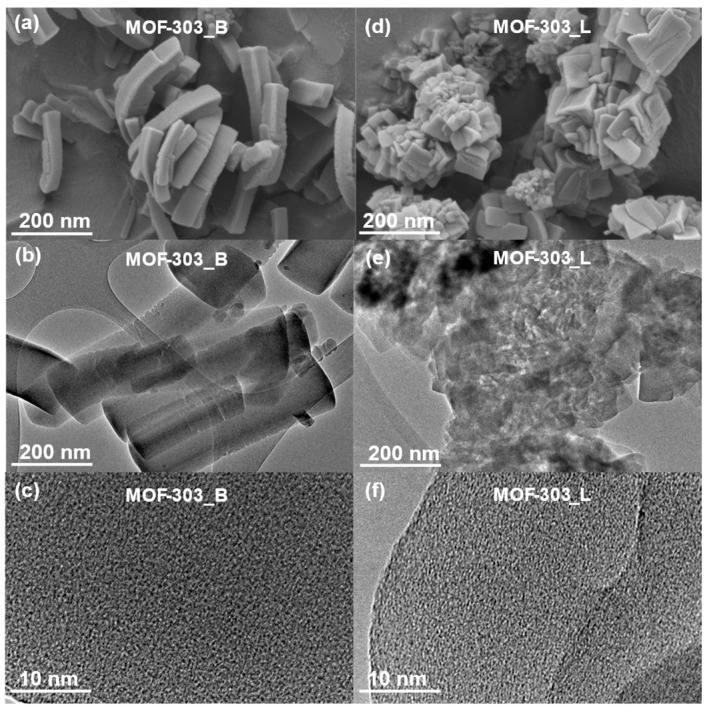
SEM (**a**,**d**) and TEM (**b**,**c**,**e**,**f**) images of MOF-303_B and MOF-303_L materials, respectively.

**Figure 3:**
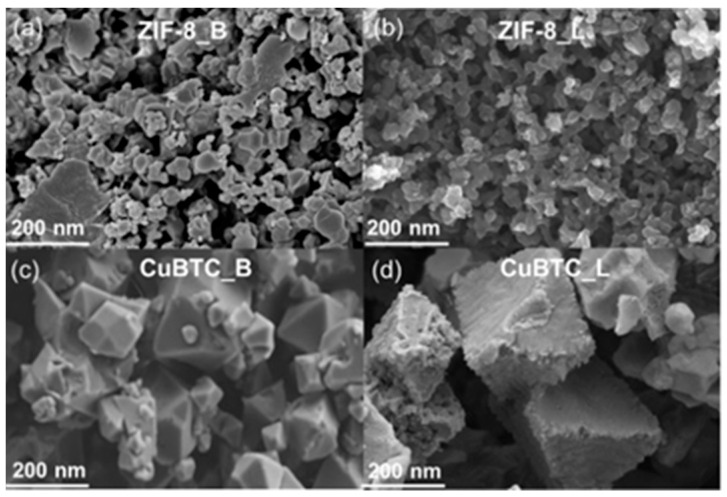
SEM images of ZIF-8 (**a**,**b**) and CuBTC (**c**,**d**) prepared via liquid (L) and ball milling methods (B).

Figure 2 and Figure 3 show the morphologies of the MOFs studied. Depending on the synthetic method, MOF-303 crystals with a different morphology were obtained, as can be seen in the scanning electron microscope (SEM) images (Figure 2a,d). Rod-shaped crystals of MOF-303 were formed under ball milling conditions (MOF-303_B), whereas cubic-shaped crystals appeared in the case of the solution-based method (MOF-303_L). The straight rods of MOF-303_B bent under voltage when the SEM images were taken. The transmission electron microscope (TEM) images show a highly developed porous structure of both materials MOF-303_B (Figure 2b,c) and MOF-303_L (Figure 2e,f). ZIF-8 particles are roughly spherical, but some ZIF-8_B crystals are polyhedral [61,62], while the CuBTC samples show an octahedral morphology [63]; see Figure 3. Strong crystal agglomeration is observed for all presented MOFs.

**Figure 4:**
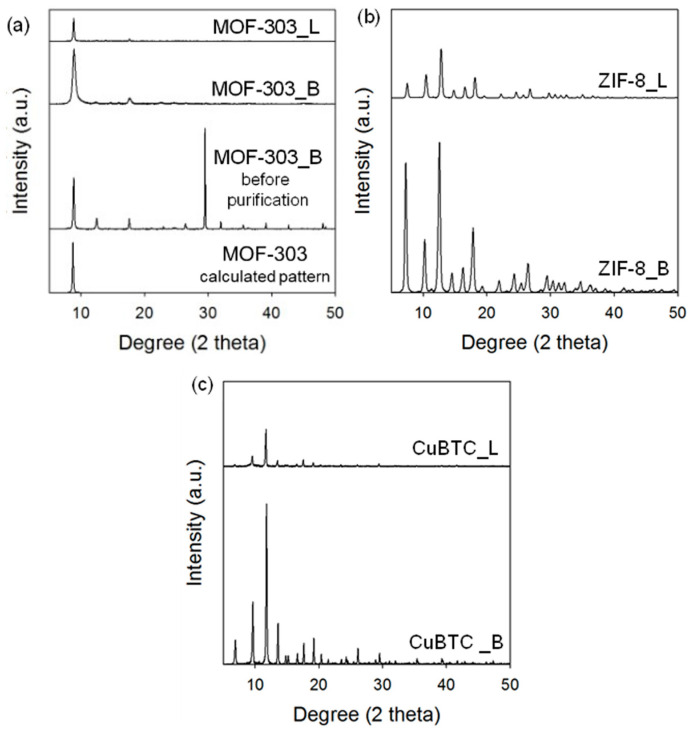
XRD patterns of (**a**) MOF-303, (**b**) ZIF-8, and (**c**) CuBTC prepared via solvothermal (L) and mechanochemical methods (B).

Figure 4 shows diffractograms of the MOFs obtained by mechanochemical and solvothermal methods. The X-ray diffraction (XRD) measurements confirm the structure and crystallinity of the MOFs; the patterns are in line with those reported in [5,64,65]. As can be seen, the intensity of the peaks on the XRD patterns of the MOFs synthesized via the mechanochemical method is higher, implying their higher crystallinity compared to their counterparts obtained by using the solution-based methods. Apparently, the high-energy ball milling affords a more uniform dispersion of reagents, which results in more homogeneous MOF structures, hence having higher crystallinity compared to the solvothermal methods. Figure 4a shows the diffractogram of the MOF-303 sample immediately after the ball milling of substrates without the purification step. In this case, peaks originating from organic linkers are also visible in the pattern. The diffractogram of purified MOF-303 shows only intensive peaks derived from the MOF crystals, confirming the necessity of the purification step.

**Figure 5:**
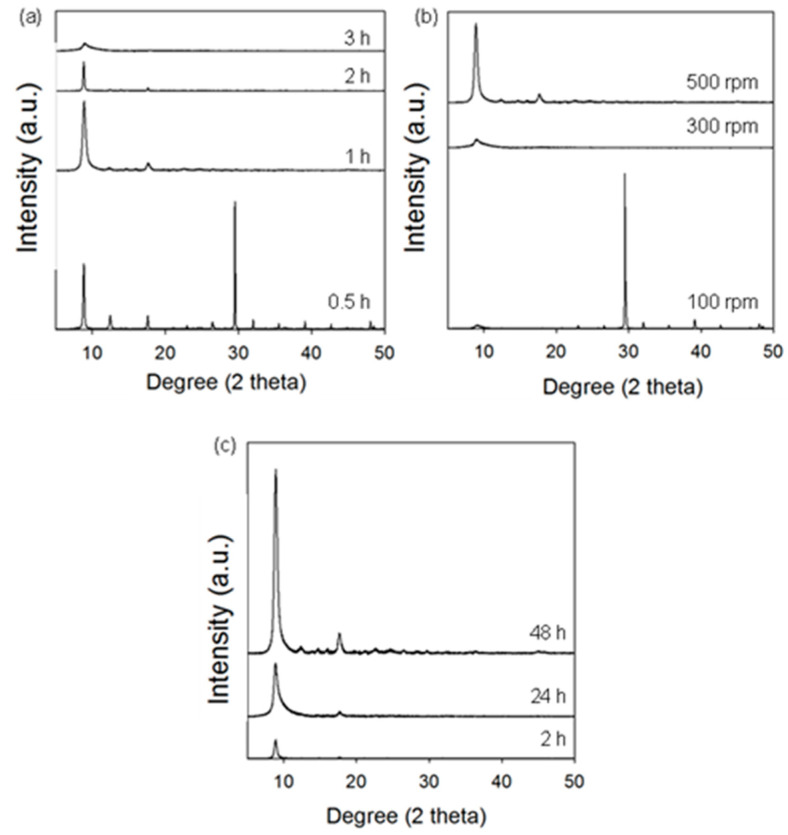
XRD patterns of MOF-303_B samples obtained under different conditions by changing (**a**) ball milling time, (**b**) rotational speed, and (**c**) purification time.

Figure 5 shows the diffractograms of the samples obtained according to the procedure used for MOF-303_B but by changing one selected parameter, i.e., grinding time, grinding speed, or purification time. The optimal ball milling conditions that afford MOF-303 with the highest porosity are as follows: 1 h of ball milling at a rotational speed of 500 rpm and a purification time of 48 h. In turn, 30 min of ball milling is insufficient for MOF-303 crystallization, and the corresponding diffractogram reveals only peaks originating from the organic linker. On the other side, extending the milling time to 2 and 3 h results in a relatively low intensity of the MOF-303 peaks, which may be attributed to the intensification of crystal defects during the long-time milling (Figure 5a). Different speeds of milling were also examined. The MOF-303 structure was not formed while milling was conducted for 1 h at a lower grinding speed of 100 rpm. Using a higher milling speed of 300 rpm was also insufficient for the synthesis reaction; one low-intensity peak appears in the corresponding XRD pattern (Figure 5b). As can be seen in Figure 5c, shortening the purification time results in lower intensities of MOF peaks, and thus, purification within 48 h in methanol is necessary to obtain pure MOF. Moreover, the gradually increasing intensity of the XRD peaks, along with the extended time of soaking in methanol, implies that the crystallization and growth of MOF crystals may further occur during the purification process.

**Figure 6:**
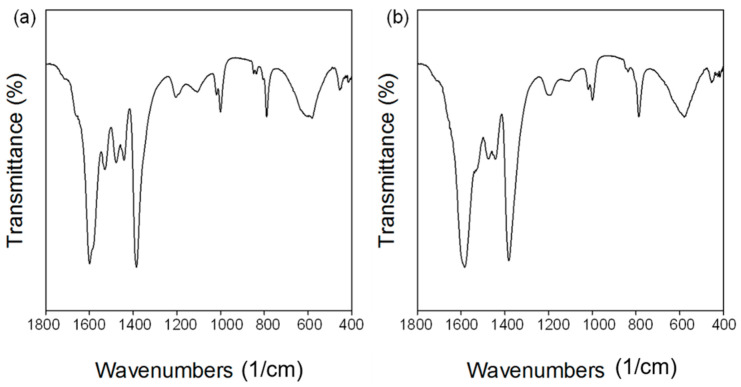
FTIR spectra of (**a**) MOF-303_B and (**b**) MOF-303_L.

The chemical bonding of MOF-303 was studied by Fourier-transform infrared (FTIR) spectroscopy in the range of 1800–400 1/cm (Figure 6). The FTIR spectra show absorption bands at 1600 and 1386 1/cm for MOF-303_B and 1584 and 1382 1/cm for MOF-303_L, which confirm the binding of carboxyl groups of the organic linker with aluminum atoms (COO-Al bond) [30]. The absorption bands corresponding to the N-NH bond, C-C bond, and C=N bond are observed for both MOFs at around 1001, 1478, and 1531 1/cm, respectively [30].

**Figure 7:**
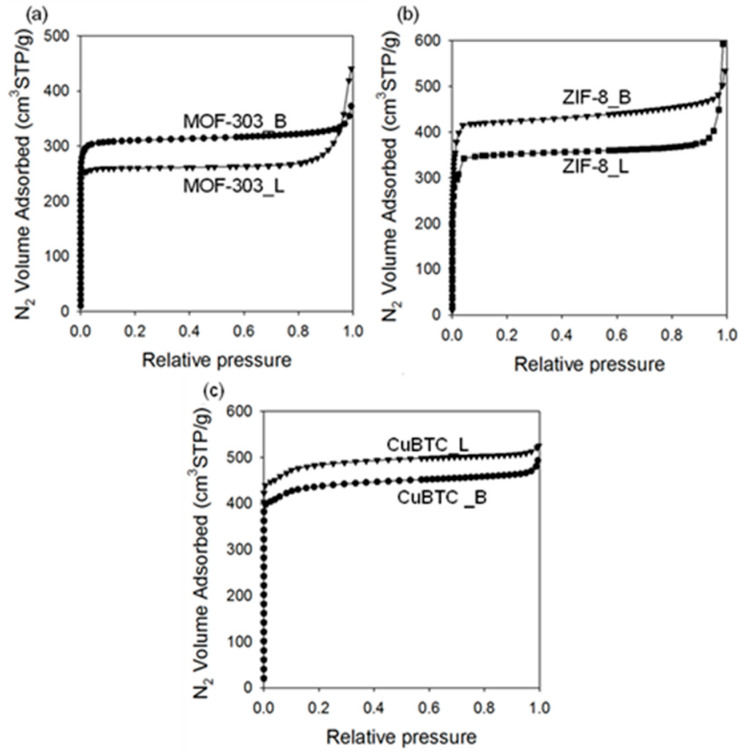
Nitrogen adsorption isotherms measured at −196 °C for (**a**) MOF-303, (**b**) ZIF-8, and (**c**) CuBTC prepared via solvothermal (L) and mechanochemical methods (B).

**Figure 8:**
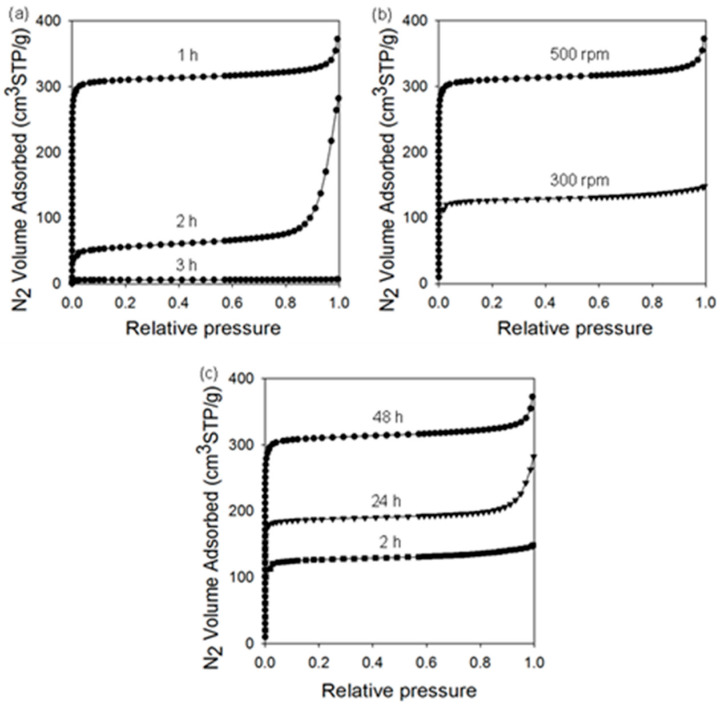
Nitrogen adsorption isotherms measured at −196 °C on MOF-303_B samples obtained under different conditions by changing (**a**) ball milling time, (**b**) rotational speed, and (**c**) purification time.

**Figure 9:**
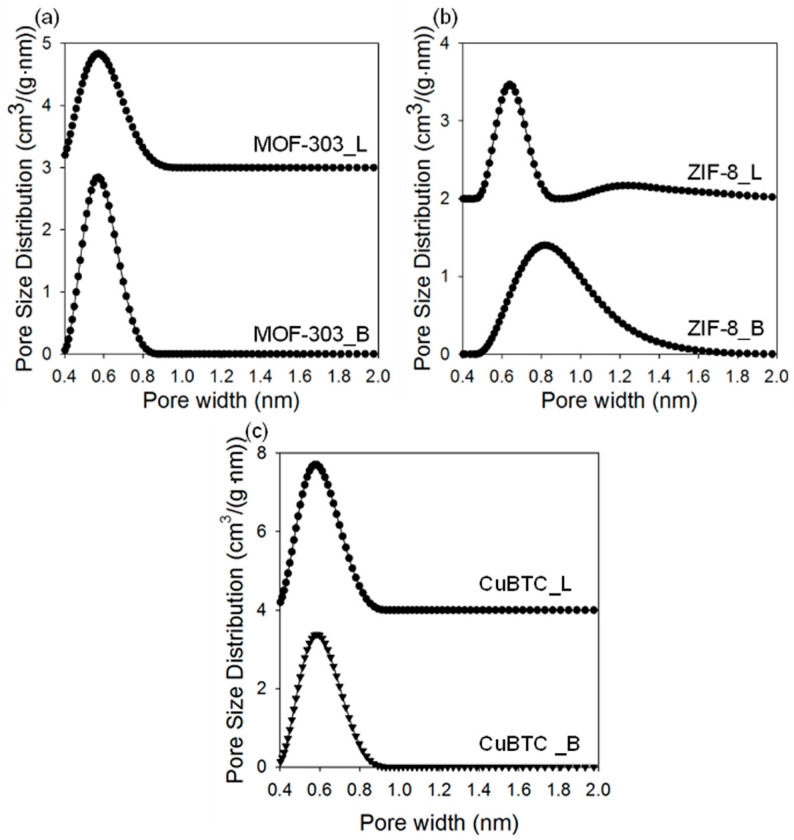
Pore size distribution functions for (**a**) MOF-303, (**b**) ZIF-8, and (**c**) CuBTC prepared via solvothermal (L) and mechanochemical methods (B).

Analysis of low-temperature nitrogen adsorption isotherms was used to determine structural parameters, such as the specific surface area, total pore volume, and pore size distribution. Figure 7, Figure 8 and Figure 9 show nitrogen adsorption isotherms measured at −196 °C and the corresponding PSD curves determined for all samples studied. The measured nitrogen isotherms for all samples studied are I-type, according to the IUPAC classification, which is characteristic of microporous materials [66]. In some cases, adsorption at higher relative pressures increases, as in the case of type II isotherms, indicating a higher external surface area associated with larger crystals and/or agglomerates. MOF-303_B shows a high SSA of 1180 m^2^/g and V_t_ of 0.58 cm^3^/g. For comparison, the SSA value of the sample prepared using the solvothermal method is lower (994 m^2^/g), and its pore volume V_t_ is higher (0.69 m^2^/g). The average pore size in both samples is around 0.6 nm. Interestingly, the mechanochemically synthesized MOFs show higher SSAs than their counterparts synthesized using solution-based methods (except CuBTC). For example, the differences in the values of SSAs depend on the MOF and are about 18.7% for MOF-303, 27.7% for ZIF-8, and 9.5% for CuBTC. These differences in the specific surface areas of the MOFs obtained by two distinct methods may be associated with a divergent (i) dispersion/dissolution of reactants, (ii) diffusion and contact of reagents, (iii) structure formation processes, and (iv) product stability during synthesis. Mechanochemical procedures, characterized by vigorous agitation and/or the comminution of substrates, may afford a more uniform dispersion of the reactants (especially while using hardly soluble ones), yielding more homogeneous MOF structures compared to the solvothermal conditions. This process was favorable in the syntheses of both MOF-303_B and ZIF-8_B, which showed increased porosity and higher crystallinity, as evidenced by XRD analysis, compared to the samples obtained solvothermally (MOF-303_L and ZIF-8_L). Conversely, in the case of CuBTC, such high-energy mechanical action led to diminished porosity compared to its solvothermally obtained counterpart, apparently due to structural damage upon milling, which is associated with its relatively low stability.

An inverse relationship is observed for the V_t_ values, which are higher for the samples obtained via the solvothermal methods, of about 17.2% for MOF-303, 16.9% for ZIF-8, and 6.6% for CuBTC (see Table 1). Ball milling conditions have a significant effect on the porosity of the resulting MOF-303 samples. Both the extension of the milling time and reduction in the rotational speed led to the samples with lower SSAs, i.e., 204 m^2^/g or 24 m^2^/g for the MOF-303 prepared by milling substrates for 2 or 3 h, respectively, and 479 m^2^/g for the MOF-303 prepared by milling substrates at a rotational speed of 300 rpm. The extended milling durations (2 h and 3 h) probably induced the structural degradation of MOF-303, which was already formed after 1 h of milling, leading to a significant reduction in its crystallinity and porosity. Shortening the purification time to 24 h or 2 h affords samples with lower SSA values of 713 m^2^/g and 446 m^2^/g, respectively.

### 2.2. CO_2_ Adsorption

**Figure 10:**
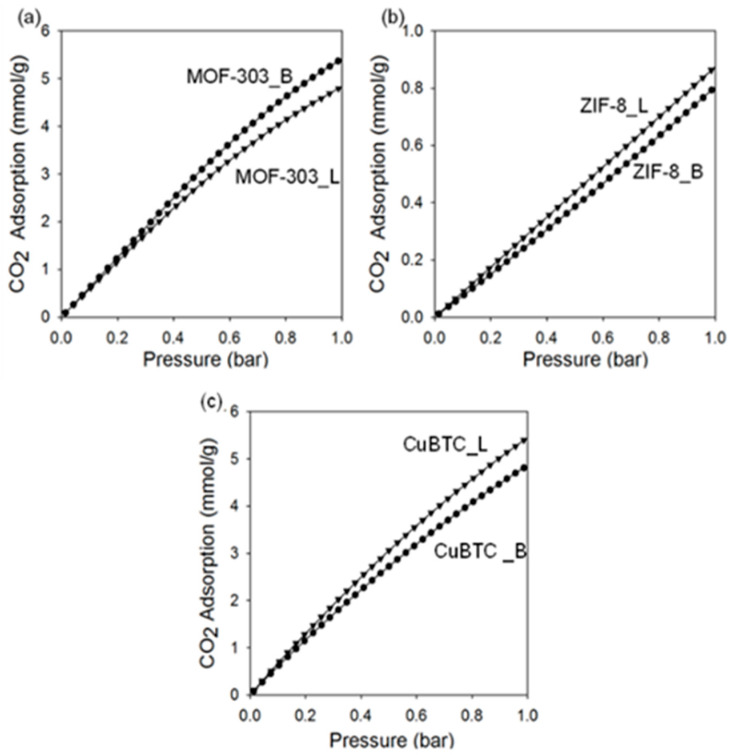
CO_2_ adsorption isotherms measured at 25 °C on (**a**) MOF-303, (**b**) ZIF-8, and (**c**) CuBTC prepared via solvothermal (L) and mechanochemical methods (B).

**Figure 11:**
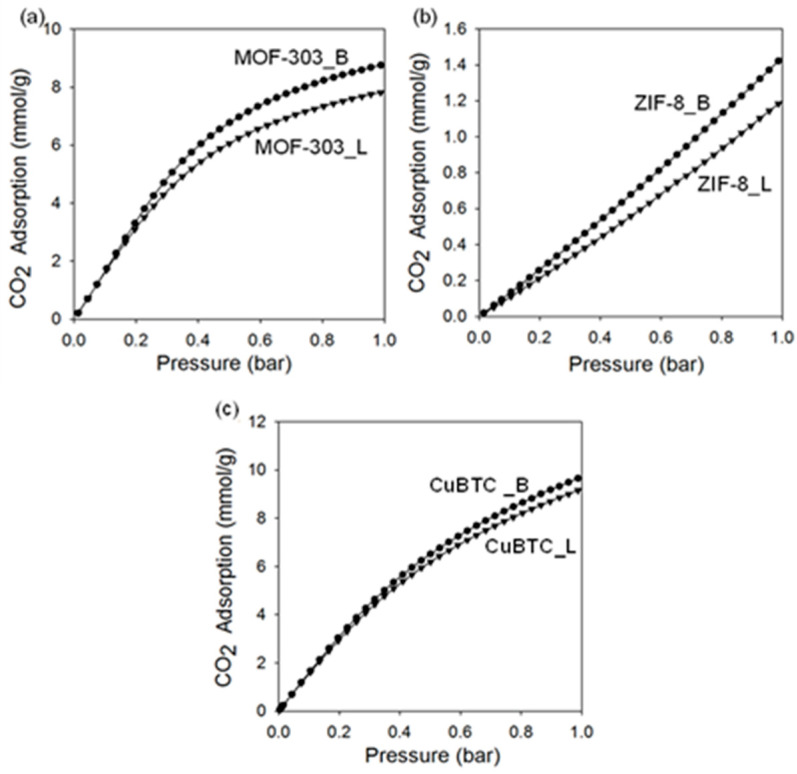
CO_2_ adsorption isotherms measured at 0 °C on (**a**) MOF-303, (**b**) ZIF-8, and (**c**) CuBTC prepared via solvothermal (L) and mechanochemical methods (B).

Figure 10 and Figure 11 show the carbon dioxide adsorption isotherms at 25 °C and 0 °C up to 1 bar. The MOF-303 that was prepared under the ball milling conditions adsorbed the highest amount of CO_2_, reaching 9.5 mmol/g at 0 °C and 5.5 mmol/g at 25 °C under 1 bar. Lower CO_2_ adsorption capacities under the same conditions are observed for MOF-303_L, namely, 8.0 mmol/g and 4.9 mmol/g, respectively (Table 2). The results are comparable to those reported in the literature, e.g., the CO_2_ adsorption capacity of 5.1 mmol/g at 25 °C and under 1 bar is reported for the MOF-303 prepared via a solution-based method [22]. The high CO_2_ adsorption capacity of MOF-303 is attributed to its high specific surface area and small pore size, as well as the intermolecular interactions with adsorption sites, such as μ-OH, N-H, N-N, and C-H, which originate from the organic linker used [22]. The enhanced adsorption properties of MOF-303_B in comparison to MOF-303_L are associated with a larger volume of ultramicropores (V_ultra_ = 0.54 cm^3^/g)_,_ higher crystalline order and, hence, more accessible adsorption sites in the mechanochemically prepared MOF. The obtained results indicate that the applied synthesis method highly influences the structure and properties of the final product. For instance, ball milling introduces defects into the crystalline structure of MOFs, creating additional active sites for CO_2_ adsorption. According to the literature, MOF-303 shows high stability during cyclic CO_2_ adsorption–desorption measurements, as evidenced by 50 cycles without measurable material degradation or noticeably reduced adsorption properties [22].

CuBTC is a well-known MOF with a very high CO_2_ adsorption capacity of up to 8–9 mmol/g at 0 °C and under 1 bar [67,68,69], and thus, it was selected for comparison. CuBTC MOFs prepared in this work, i.e., CuBTC_B and CuBTC_L, show comparable CO_2_ adsorption capacities of 4.9 mmol/g and 5.5 mmol/g at 25 °C and under 1 bar and 9.1 mmol/g and 9.6 mmol/g at 0 °C and under 1 bar, respectively. The slightly lower CO_2_ adsorption on CuBTC_B was anticipated due to its lower porosity, which is attributed to its relatively low stability and, therefore, partial structural damage and pore collapse caused by milling.

Theoretical [70] and experimental [71] investigations have shown a strong affinity of water molecules to copper centers in the CuBTC framework. Sensitivity to water (hydrophilicity) could be a major weakness of CuBTC as an adsorbent that limits or disables its practical application. Conversely, MOF-303 shows high hydrolytic stability, i.e., it retains its structure and properties in the presence of water. For instance, it was confirmed by carrying out 150 cycles of water adsorption and desorption without measurable material degradation [15]. Therefore, MOF-303 can be considered an effective and sufficiently stable CO_2_ adsorbent for practical uses.

ZIF-8 MOFs adsorb CO_2_ in rather small amounts. To increase their CO_2_ adsorption capacities, structure modifications are necessary, e.g., introducing functional groups or heteroatoms. Moreover, some mechanochemically synthesized metal–organic structures show higher CO_2_ adsorption capacities compared to those synthesized solvothermally, probably due to a higher contribution of micropores in their structures, especially those with sizes smaller than 0.7 nm, i.e., ultramicropores (Table 1).

Thus far, not many papers have been reported on MOF-303 presenting various synthesis methods in solutions that led to MOFs with different SSAs, most often in the range from about 900 to about 1500 m^2^/g; see Table 3. Our novel synthesis method, based on ball milling, afforded MOF-303 with a comparable SSA of 1180 m^2^/g and a very high CO_2_ adsorption capacity, which is superior in considering favorable synthesis conditions.

In general, one of the main concepts of mechanochemical synthesis is to eliminate the use of solvents, which minimizes chemical wastes and reduces environmental pollution and production costs. However, in the synthesis of MOF-303, the subsequent purification process in organic solvents is very important and necessary to obtain pure crystals of sufficiently good quality, as in the case of solvothermal synthesis.

One should note the other advantages of mechanochemical synthesis, as it usually takes from a few minutes to several hours, which is faster than typical solvothermal synthesis, which often lasts much longer. Moreover, ball milling is carried out without applying external heating of the reaction mixture or increased pressure, unlike typical solvothermal methods, which results in lower energy consumption and lower costs. Therefore, such synthesis conditions are considered safe and in line with promoting sustainable chemical processes.

## 3. Materials and Methods

### 3.1. Chemicals

MOF-303 was prepared using 1H-pyrazole-3,5-dicarboxylic acid hydrate (H_2_PZDC·H_2_O, 97%, AmBeed, Arlington Heights, IL, USA), aluminum nitrate nonahydrate (>99%, POCH S.A., Boston, MA, USA), sodium hydroxide (98.8%, POCH S.A.), and methanol (99.8%, POCH S.A.).

ZIF-8 was obtained using zinc oxide (99%, Sigma-Aldrich, St. Louis, MO, USA), zinc acetate dihydrate (99.9%, POCH), 2-methylimidazole (99%, Sigma-Aldrich), and *N*,*N*′-dimethyloformamid (99.5%, Acros Organics, Geel, Belgium).

CuBTC (also known as HKUST-1) was synthesized using copper nitrate trihydrate (99%, Sigma-Aldrich), zinc oxide (99%, Sigma-Aldrich), 1,3,5-benzenetricarboxylic acid (H_3_BTC, 95%, Sigma-Aldrich), and ethanol (99.8%, POCH).

All chemicals were analytically pure and used without further purification.

### 3.2. Synthesis of MOFs

#### 3.2.1. Mechanochemical Synthesis of MOF-303

**Figure 12:**
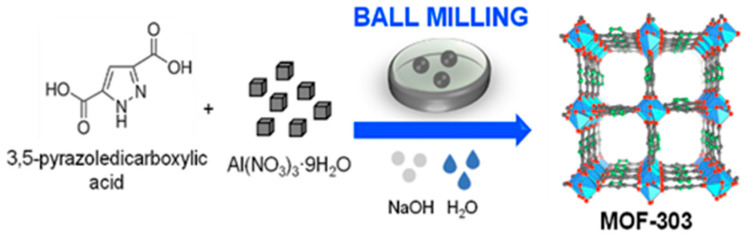
Scheme illustrating mechanochemical synthesis of MOF-303 [5]. MOF-303 structure was adapted with permission from ref. [5]. C—gray, N—green, O—red, Al—blue polyhedra. Copyright © 2018 Creative Commons Attribution-NonCommercial License 4.0 (CC BY-NC).

In brief, 0.75 g of 3,5-pyrazoledicarboxylic acid, 0.26 g of NaOH, and 2 mL of deionized water were initially milled for 10 min. Then, 1.62 g of Al(NO_3_)_3_·9H_2_O was added, and ball milling was continued for 0.5 h (at 500 rpm), 1 h (at 100, 300 or 500 rpm), 2 h (at 500 rpm), or 3 h (at 500 rpm). Next, the paste was washed with water and methanol at different times (2 h, 24 h or 48 h) and dried in the air. These different conditions were used to optimize the mechanochemical synthesis of MOF-303. Further, the product was activated under vacuum at room temperature for 6 h. After that, the MOF sample was heated at 100 °C for 6 h and then at 150 °C for another 6 h. The molar ratio of the reactants and the purification procedure were adopted from ref. [26]. The MOF-303 sample that was synthesized under optimal conditions (ball milling for 1 h at 500 rpm, purification for 48 h) was denoted as MOF-303_B, and the reference samples were described with additional information in brackets (e.g., 2 h ball milling; 300 rpm; 24 h purification). A schematic illustration of the mechanochemical synthesis of MOF-303 is shown in Figure 12.

#### 3.2.2. Solvothermal Synthesis of MOF-303

MOF-303 was prepared using the method reported by Wang et al. [26]. A total of 0.75 g of 3,5-dicarboxylic acid and 0.26 g of NaOH were dissolved in deionized water (75 mL). The resulting mixture was sonicated for 10 min and heated for 30 min in a preheated oven at 100 °C until the solution was clear. Afterward, 1.62 g of Al(NO_3_)_3_·9H_2_O was added to the solution. The reaction mixture was placed in a preheated oven at 100 °C and kept for 15 h. The purification and activation procedures were the same as above. The sample was denoted as MOF-303_L.

#### 3.2.3. Mechanochemical Synthesis of ZIF-8

A total of 0.407 g of ZnO and 1.10 g of Zn(CH_3_COO)_2_ were milled for 10 min (500 rpm). Then, 2.465 g of 2-methyloimidazolate was added, and milling continued for 60 min. Next, the paste was washed with DMF and MeOH, dried at 70 °C for 24 h, and activated at 150 °C for 2 h. The molar ratio of the reactants and the purification procedure were adopted from ref. [73]. The resulting sample was denoted as ZIF-8_B.

#### 3.2.4. Solvothermal Synthesis of ZIF-8

ZIF-8 was prepared by using the hydroxy double salts (HDS) method reported by Zhao et al. [73]. First, 0.293 g of ZnO and 8 mL of deionized water were sonicated for 10 min to form a slurry, which was mixed with 5 mL of aqueous Zn(CH_3_COO)_2_ (1.1 g) and 5 mL DMF for 24 h at room temperature. Then, 3 mL of the obtained suspension was added to 9 mL of the 2-methylimidazole (2.465 g) DMF solution under magnetic stirring at room temperature for 10 min. The product was filtered, washed several times with small portions of DMF, and activated at 150 °C for 2 h. The sample was denoted as ZIF-8_L.

#### 3.2.5. Mechanochemical Synthesis of CuBTC

CuBTC was prepared using the method reported by Szczęśniak et al. [67]. A total of 1.74 g of Cu(NO_3_)_2_·3H_2_O and 0.293 g of ZnO were milled for 30 min (500 rpm). Then, 0.84 g of 1,3,5-benzenetricarboxylic acid was added, and milling was continued for 30 min. Next, the paste was washed with EtOH, vacuum dried at room temperature overnight, washed with an ethanol–water mixture, dried at room temperature for 24 h, and activated at 150 °C for 6 h. The sample was denoted as CuBTC_B.

#### 3.2.6. Solvothermal Synthesis of CuBTC

CuBTC was prepared using the HDS method reported by Zhao et al. [73]. First, 0.293 g of ZnO and 8 mL of deionized water were sonicated for 10 min to form a slurry. Meanwhile, two separate solutions were prepared by dissolving 1.74 g of Cu(NO_3_)_2_·3H_2_O in 8 mL of deionized water and dissolving 0.84 g of 1,3,5-benzenetricarboxylic acid (H_3_BTC) in 16 mL of ethanol. Then, the ZnO slurry was mixed with 16 mL of DMF. Next, the Cu(NO_3_)_2_ aqueous solution and H_3_BTC ethanolic solution were successively added under magnetic stirring. After 30 min of reaction, the product was promptly filtered and washed with EtOH. The product was dried in a fume hood overnight, then washed with an ethanol–water mixture, again dried at room temperature for 24 h, and activated at 150 °C for 6 h. The sample was denoted as CuBTC_L.

HDS compounds are formed by the reaction of a divalent metal oxide with a different divalent cation and exhibit excellent anion exchange properties. For instance, in the synthesis of CuBTC, ZnO is essential to produce (Zn and Cu) hydroxy nitrate HDS, which can further rapidly react with ligand anions to form CuBTC crystals, accelerating the synthesis at room temperature.

All ball milling syntheses were performed using a Planetary Mono Mill PULVERISETTE 6 classic line ball milling machine (Fritsch, Bahnhofstraße, Germany), equipped with a 45 mL ceramic milling bowl and 8 ceramic balls of 1 cm in diameter.

The yield of the MOF synthesis is an important factor and strongly depends on the method. In this study, the synthesis yield of MOFs through ball milling was around 50–60%, whereas, using solvothermal methods, higher yields of up to 80% were achieved. The values depend on specific experimental conditions, the type of MOF, and other factors influencing the synthesis process.

### 3.3. Measurements and Calculations

X-ray diffraction analysis, nitrogen, and CO_2_ adsorption were used to determine the crystallographic phases, porous structure, and CO_2_ adsorption properties of all samples, respectively. Additionally, SEM and TEM images were performed for the selected samples. The SEM images were taken using a scanning electron microscope, the LEO 1530 manufactured by Zeiss (Oberkochen, Germany), at a 2 kV acceleration voltage. The TEM images were carried out on a Titan G2 60–300 kV microscope operating at 300 kV, manufactured by FEI (Hillsboro, OR, USA). The XRD analysis was conducted using the Bruker D2 PHASER diffractometer with CuKα X-rays operating at 30 V and 10 mA, in the range of 3° < 2 θ < 70° at room temperature. The FTIR-ATR (ATR, attenuated total reflectance) spectra were obtained on a FTIR Nicolet 8700A spectrometer manufactured by Thermo Scientific (Waltham, MA, USA). Nitrogen adsorption isotherms were measured at −196 °C using an ASAP 2020 volumetric analyzer manufactured by Micromeritics Instrument Corp. (Norcross, GA, USA). Carbon dioxide isotherms were measured at 0 °C and 25 °C. All samples were outgassed for 12 h at 150 °C prior to the adsorption measurements. The purities of the N_2_ and CO_2_ gases used in the adsorption measurements were 99.999% and 99.99%, respectively. The SSA was calculated from the low-temperature nitrogen adsorption data in a relative pressure range of 0.05 < *p*/*p*_0_ < 0.2 using the Brunauer–Emmett–Teller method [74]. The V_t_ was calculated using the volume of liquid nitrogen adsorbed at a relative pressure of ~0.99. The pore size distribution (PSD), micropore volume (V_micro_), and ultramicropore volume (V_ultra_) were calculated from low-temperature nitrogen adsorption data using the NLDFT method developed for adsorption and capillary condensation in cylindrical pores [75,76]. The calculations were performed using the numerical program SAIEUS. The mesopore volume (V_meso_) was calculated as the difference between V_t_ and V_micro_.

## 4. Conclusions

The presented results reveal that fast ball milling could be successfully used to conduct chemical reactions between metal salt and organic linker, forming MOF-303 crystals. The reported method enables us to shorten the synthesis time and significantly reduces the volume of solvents needed in a typical solvent-based preparation of MOF-303. The presented strategy meets the green chemistry conception and has a high potential for practical uses. The MOF-303 sample that was synthesized mechanochemically exhibits a high CO_2_ adsorption capacity of 9.5 mmol/g at 0 °C and 5.5 mmol/g at 25 °C and under 1 bar; thus, it is very promising for efficient CO_2_ adsorption. A series of diverse MOFs synthesized via the mechanochemical method show both comparable (or better) porosity and CO_2_ adsorption capacities than the corresponding MOFs obtained using the solution-based methods. Although mechanochemical synthesis of MOFs is an environmentally friendly method, their post-synthesis purification may require solvents and generate some liquid waste, but this step is analogous to the case of solvothermal synthesis.

## Figures and Tables

**Table 1:** Structural parameters evaluated from N_2_ adsorption data for the MOF samples studied.

Sample	SSA [m^2^/g]	V_t_ [cm^3^/g]	V_micro_ [cm^3^/g]	V_ultra_ [cm^3^/g]
MOF-303_B	1180	0.58	0.58	0.54
MOF-303_B (2 h ball milling)	204	0.44	0.09	0.06
MOF-303_B (3 h ball milling)	24	0.01	0.01	0.01
MOF-303_B (300 rpm)	479	0.23	0.23	0.18
MOF-303_B (24 h purification)	713	0.44	0.36	0.08
MOF-303_B (2 h purification)	446	0.69	0.19	0.14
MOF-303_L ZIF-8_B	994	0.68	0.48	0.40
	1609	0.83	0.70	0.10
ZIF-8_L	1260	0.97	0.50	0
CuBTC_B	1638	0.84	0.76	0.69
CuBTC_L	1809	0.93	0.81	0.77

**Table 2:** CO_2_ adsorption at 0 °C and 25 °C under 1 bar for the MOF samples studied.

Sample	CO_2_ Adsorption [mmol/g]	
	25 °C, 1 bar	0 °C, 1 bar
MOF-303_B	5.5	9.5
MOF-303_L	4.9	8.0
ZIF-8_B	0.8	1.5
ZIF-8_L	0.7	1.2
CuBTC_B	4.9	9.1
CuBTC_L	5.5	9.6

**Table 3:** Structural parameters and application of MOF-303 prepared via different methods.

Synthesis Method	SSA [m^2^/g]	V_t_ [cm^3^/g]	Application	Ref.
Ball milling	1180	0.60	CO_2_ adsorption: 5.5 mmol/g (25 °C, 1 bar) 9.5 mmol/g (0 °C, 1 bar)	This work
Solvothermal	1529	0.55	CO_2_ adsorption: 5.1 mmol/g (25 °C, 1 bar)	[22]
	1372	0.52	Atmospheric water harvesting	[11]
	1355	0.52	Atmospheric water harvesting	[8]
	1343	-	Xenon adsorption	[26]
	1342		Atmospheric water harvesting	[12]
	1292	-	Ammonia adsorption	[24]
	1119	-	Atmospheric water harvesting	[6]
	1046	-	Fe^3+^ adsorption	[30]
	989	-	Atmospheric water harvesting	[5]
	910	-	Transition metals adsorption	[32]
	887	-	Desalination	[31]
Reflux	1392	0.52	Atmospheric water harvesting	[11]
	1384	-	Atmospheric water harvesting	[12]
	1374	0.52	Atmospheric water harvesting	[11]
Microwave	1343	-	C_2_H_2_ adsorption	[25]
	1307		Atmospheric water harvesting	[12]
	658	-	Proton conduction material	[52]
Vessel	1380		Atmospheric water harvesting	[12]
In situ growth on polymeric nanofibers	426	-	Particulate matter adsorption	[28]
Other/non-stated	1212	0.58	SO_2_ adsorption	[23]
	917 (MIL-160(Al)/MOF-303)	0.44	Atmospheric water harvesting	[16]
	845 (GO@Fe_3_O_4_@MOF-303)	-	-	[72]

## Data Availability

The data presented in this study are available in article.
